# Pre‐diagnostic levels of sVEGFR2, sTNFR2, sIL‐2Rα and sIL‐6R are associated with glioma risk: A nested case–control study of repeated samples

**DOI:** 10.1002/cam4.4505

**Published:** 2022-01-14

**Authors:** Wendy Yi‐Ying Wu, Florentin Späth, Carl Wibom, Benny Björkblom, Anna M. Dahlin, Beatrice Melin

**Affiliations:** ^1^ Department of Radiation Sciences, Oncology Umeå University Umeå Sweden; ^2^ Department of Chemistry Umeå University Umeå Sweden

**Keywords:** a nested case–control study, cytokines, glioma aetiology

## Abstract

No strong aetiological factors have been established for glioma aside from genetic mutations and variants, ionising radiation and an inverse relationship with asthmas and allergies. Our aim was to investigate the association between pre‐diagnostic immune protein levels and glioma risk. We conducted a case–control study nested in the Northern Sweden Health and Disease Study cohort. We analysed 133 glioma cases and 133 control subjects matched by age, sex and date of blood donation. ELISA or Luminex bead‐based multiplex assays were used to measure plasma levels of 19 proteins. Conditional logistic regression models were used to estimate the odds ratios and 95% CIs. To further model the protein trajectories over time, the linear mixed‐effects models were conducted. We found that the levels of sVEGFR2, sTNFR2, sIL‐2Rα and sIL‐6R were associated with glioma risk. After adjusting for the time between blood sample collection and glioma diagnosis, the odds ratios were 1.72 (95% CI = 1.01–2.93), 1.48 (95% CI = 1.01–2.16) and 1.90 (95% CI = 1.14–3.17) for sTNFR2, sIL‐2Rα and sIL‐6R, respectively. The trajectory of sVEGFR2 concentrations over time was different between cases and controls (*p*‐value = 0.031), increasing for cases (0.8% per year) and constant for controls. Our findings suggest these proteins play important roles in gliomagenesis.

## Graphical abstract

In a nested case–control study, we found that the pre‐diagnostic levels of sVEGFR2, sTNFR2, sIL‐2Rα and sIL‐6R were associated with glioma risk. Our findings suggest these proteins play important roles in gliomagenesis.

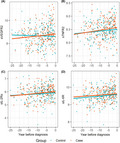

## 1 INTRODUCTION

Gliomas, the most frequent primary brain tumours, include glioblastoma (GBM), the most common and aggressive subtype with a median overall survival of 14–17 months. However, only a few risk factors for glioma have been identified, including genetic predisposition and high doses of ionising radiation.^1^, ^2^ Thus, glioma aetiology remains unknown for the majority of patients. Previous studies have shown an inverse relationship between asthma and allergies and glioma risk, potentially indicating subtle immune dysregulation is involved in glioma development.^3^ Studies over the past two decades have provided important information on the role of the immune system in gliomagenesis.^4^ As cytokines are chemical messengers that regulate the innate and adaptive immune systems, the interaction with their receptors which can affect proliferation, angiogenesis and aggressiveness––might play an important role in glioma progression.^5^, ^6^ Interestingly, haematological malignancies and glioma have some common aetiological pathways, such as exposure to ionising radiation and an inverse relationship with asthma and allergies.^7^ In lymphoma studies, analysis of several relevant proteins, including sCD23, sCD27, sCD30 and CXCL13, in pre‐diagnostic plasma samples have shown a significant difference between cases and controls even several years before diagnosis.^8^, ^9^ However, little is known about how the subclinical immunologic perturbations influence glioma risk. So far, only two studies investigated the association between pre‐diagnostic proteins and glioma.^10^, ^11^, ^12^ Schwartzbaum et al., who evaluated 277 serum proteins in 487 case–control sets, found that VEGF, beta‐catenin, CCL22, LIF, sIL‐10RB, IL‐4 and sIL‐4Rα were associated with glioma risk.^12^ Brenner et al., who evaluated 14 serum proteins in 457 case–control sets, found that IL‐15 and IL‐16 were associated with lower glioma risks.^10^ However, these results have not been confirmed. Here, we selected 19 immune proteins that might be involved in the development of glioma. Specifically, we evaluated the protein levels in pre‐diagnostic plasma samples from 133 glioma patients and 133 matched controls in a nested case–control study within the population‐based Northern Sweden Health and Disease Study (NSHDS). We hypothesised that altered protein levels are associated with glioma risk. To gain further insight into the patterns of immune proteins in gliomagenesis according to the time between blood sample collection and glioma diagnosis, we investigated the protein trajectories over time in cases and controls.

## 2 MATERIALS AND METHODS

### 2.1 Study population

The present study is a case–control study nested within the population‐based NSHDS cohort, which consists of three sub‐cohorts. Plasma samples used in the present study were collected from participants in two of these sub‐cohorts: the Västerbotten Intervention Programme (VIP) and Mammography Screening Project (MA). Since 1985, VIP has invited all residents of Västerbotten County to a general health check‐up at 40, 50 and 60 years of age. MA collected blood samples in connection with mammography screening visits between 1995 and 2006. The cohort consists of women aged 18–82 years, although 95% were between 48 and 70 years. Informed consent for cancer research and lifestyle diseases was collected according to the Helsinki Declaration. Glioma cases (ICD‐7 code 193 and histopathological codes 475 and 476) were identified through linkage with the Swedish Cancer Registry for the period 1982–2013. For each case, one control was randomly selected from the eligible subjects and individually matched by age (±5 months), sex and date of blood donation (±2 months). Controls were alive and free of cancer at the time of diagnosis of the matched case.

### 2.2 Protein analysis

We measured plasma concentrations of 19 immune proteins, including cytokines, chemokines, growth factors and soluble receptors. Plasma samples collected in EDTA plasma vacutainers were frozen within 1 h of blood sampling and stored at −80°C at the Medical Biobank at Umeå University. Sixteen proteins were measured on four Luminex bead‐based commercial assay panels (Millipore), including monocyte chemoattractant protein (MCP)‐3, macrophage inflammatory protein (MIP)‐1α, MIP‐1β, vascular endothelial growth factor (VEGF), fibroblast growth factor (FGF)‐2, fractalkine, transforming growth factor (TGF)‐α, interleukin (IL)‐13, tumour necrosis factor (TNF)‐α, IL‐10 (by Milliplex HCYTOMAG‐60K kit), soluble interleukin 2 receptor alpha (sIL‐2Rα), sIL‐6R, sTNFR2, sVEGFR2 (by Milliplex HSCRMAG‐32K kit), chemokine C‐X‐C motif ligand 13 (CXCL13) and sTNFR1 (by LXSAHM kit, R&D Systems). Three proteins––soluble CD23 (sCD23), sCD27 and sCD30––were measured using ELISA assays (eBioscience).

Cases and matched controls with their repeated samples were included in the same block and randomly placed on the analysis plates to minimise the plate‐mediated effect. Samples from the same case–control set were placed on the same plate, although a few samples (from eight case–control sets) were placed on separate but adjacent plates due to limited space. Samples within each block were plated at random. Laboratory analyses were performed by laboratory personnel blinded to case–control status and chronological order of the samples. The same laboratory technician performed all analyses according to the manufacturer's instructions.

Each sample was measured twice (except sCD30 due to limited plasma volume) and averaged to calculate concentrations. Table S1 shows the per cent detected above the lower limit of detection (LLOD) and coefficients of variation (CVs) of the 19 proteins.

### 2.3 Statistical analyses

First, we investigated the association between the concentration of each protein and glioma risk using conditional logistic regression model to estimate odds ratios (ORs) and 95% confidence intervals (CIs). Protein measurement was natural log‐transformed and treated as a continuous variable. Robust sandwich variances were estimated to account for correlations amongst multiple measurements from the same matched set. The sample collection time, defined as years before the date of diagnosis amongst cases and corresponding reference time for controls, was included in the model as a confounder or effect modifier. Second, to further model the protein trajectories over time, we used the linear mixed‐effects models, which accounted for the repeated measurement structure in the dataset. Models were specified with a linear term for sample collection time. Pearson correlation coefficient was used to measure the correlation between the 19 proteins. For the subjects who denoted two pre‐diagnostic samples, the sample closer to diagnosis was chosen. As glioma is highly heterogeneous, we performed a sensitivity analysis that only included GBM cases (SNOMED code 94403) and matched controls. All *p*‐values were two‐sided, and a value of 0.05 or less was considered statistically significant. Statistical analysis was performed in R version 3.6.0 (R Foundation for Statistical Computing) using the ‘survival’ and ‘lme4’ packages.^13^, ^14^

## 3 RESULTS

We identified 133 individuals diagnosed with a glioma between 1992 and 2013 who had donated one (*n* = 68) or two (*n* = 65) pre‐diagnostic blood samples to NSHDS (Table 1 and Table S2). The average age of diagnosis was 60.6 years (SD = 9.19). There were 81 (61.8%) GBM cases. The average times between blood samplings and diagnoses were 8.0, 11.8 and 4.3 years for single, first and repeated pre‐diagnostic samples, respectively. Nearly identical distribution of sex, age and sample collection times between cases and controls was evidence of the validity of the matched design (Table 1).

**TABLE 1:** Characteristics of the study subjects

Characteristics	Cases	%	Controls	%
Age at diagnosis, median (range) years	61.7 (32.5–82.7)			
SNOMED^a^, *N*				
93803 (Glioma, malignant)	3	2.3		
93823 (Glioma, mixed, oligoastrocytoma)	1	0.8		
94003 (Astrocytoma, grades I–II)	9	6.9		
94013 (Astrocytoma, anaplastic, grade III)	17	13.0		
94213 (Pilocytic astrocytoma)	4	3.1		
94403 (Glioblastoma)	81	61.8		
94423 (Pleomorphic xanthoastrocytoma)	2	1.5		
94503 (Oligodendroglioma)	9	6.9		
94513 (Oligodendroglioma, anaplastic)	5	3.8		
Time from blood sampling to diagnosis, mean years (±SD)				
Single sample	8.0 ± 5.4		8.1 ± 5.4	
First sample	11.8 ± 5.6		11.8 ± 5.6	
Repeated sample	4.3 ± 3.0		4.3 ± 3.0	
Age at sample collection, median (range) years				
Single sample	52.6 (30.3–68.4)		52.8 (30.1–68.4)	
First sample	49.9 (29.7–60.6)		50.0 (30.0–60.2)	
Repeated sample	59.6 (40.0–67.5)		59.7 (40.1–67.8)	
Sex, *N*				
Male	64	48.1	64	48.1
Female	69	51.9	69	51.9

Abbreviation: SD, standard deviation.

^a^ Information on SNOMED was missing for two cases.

**FIGURE 1:**
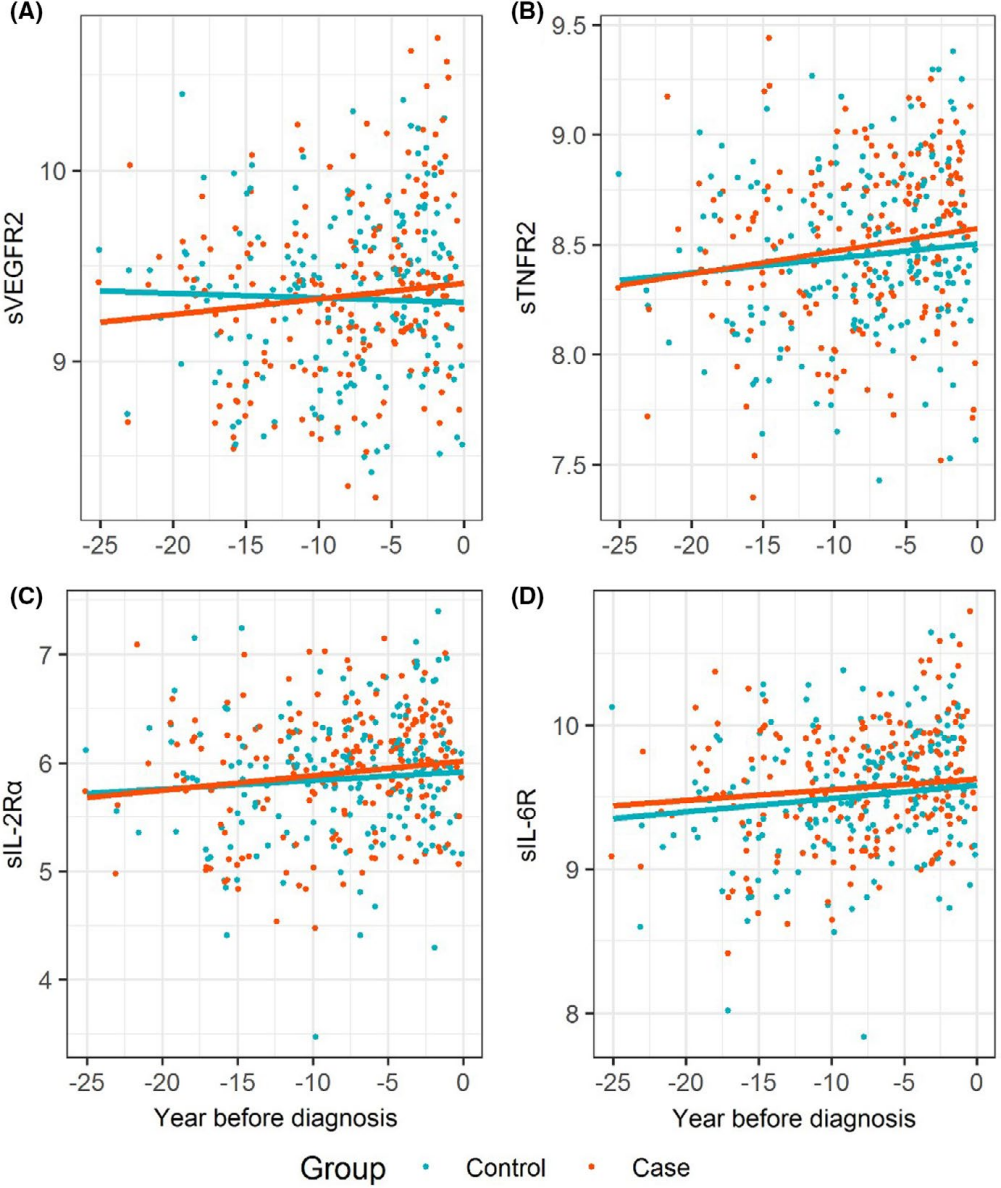
Protein changes over time in cases and controls. Protein measurements were natural log‐transformed and the regression lines were estimated from the linear‐mixed models. (A) sVEGFR2, soluble vascular endothelial growth factor receptor 2; (B) sTNFR2, soluble tumour necrosis factor receptor 2; (C) sIL‐2Rα, soluble interleukin 2 receptor alpha; (D) sIL‐6R, soluble interleukin 6 receptor

**FIGURE 2:**
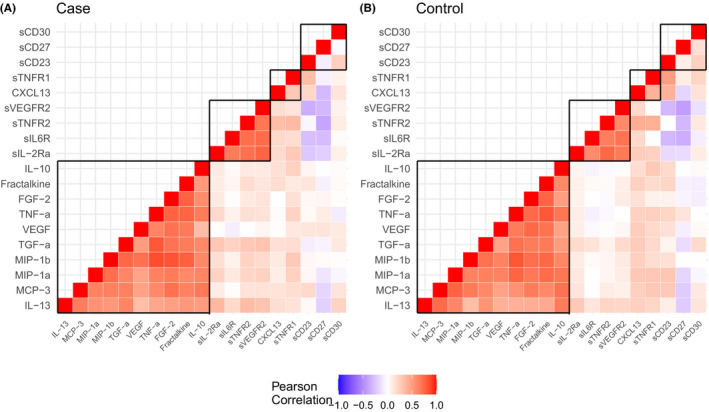
The correlation heatmap in cases (A) and controls (B). The proteins inside the boxes marked with black lines were measured from the same panel

Table 2 shows the summary statistics for the circulating protein levels in cases and controls. Of the studied proteins, statistically significant associations with risk of glioma were found for sVEGFR2, sIL‐2Rα, sTNFR2 and sIL‐6R (Table 3). The risk of glioma on sVEGFR2 varies by the sample collection time (interaction *p*‐value = 0.015). The OR for sVEGFR2 increased by a factor of 1.12 with each year to diagnosis. As shown in Figure 1A, the concentration of sVEGFR2 increased with time in cases (0.8% per year, *p*‐value = 0.02) but remained constant in controls (0.2% decrease per year, *p*‐value = 0.51). After adjusting for the sample collection time, the ORs were 1.72 (95% CI: 1.01–2.93), 1.48 (95% CI: 1.01–2.16) and 1.90 (95% CI: 1.14–3.17) for sTNFR2, sIL‐2Rα and sIL‐6R, respectively. However, there was no statistically significant difference after considering the multiple testing correction. The concentration of sTNFR2 increased with time in cases by 1.03% (*p*‐value = 0.01) but did not significantly increase in controls (0.67%, *p*‐value = 0.07) (Table S3). A similar finding was observed in sIL‐2Rα. The concentration of sIL‐2Rα increased with time significantly in cases by 1.35% (*p*‐value = 0.01) but did not significantly increase in controls (0.81%, *p*‐value = 0.11). For sIL‐6R, the concentration increased with time in controls by 0.93% per year (*p*‐value = 0.02) and 0.75% in cases (*p*‐value = 0.06). Visually, the levels of sIL‐2Rα and sTNFR2 between cases and controls started to show a difference around 15 years before the diagnosis (Figure 1B,C), and the levels of sIL‐6R were constantly higher in the cases compared with the controls (Figure 1D). However, there was no difference in the slopes of regression lines between controls and cases for sTNFR2, sIL‐2Rα and sIL‐6R. Figure 2 shows the correlation between the 19 proteins in cases and controls. Higher positive correlation between proteins was found when they was measured from the same panel. In the sensitivity analysis, which was restricted to 81 GBM cases and 81 matched controls, there were 43 (53.1%) male GBMs and the average age of diagnosis was 63.4. Two proteins––sIL‐6R (OR = 2.30, 95% CI: 1.22–4.36) and sTNFR2 (OR = 2.08, 95% CI: 1.03–4.18)––showed stronger association with GBM risk (Table S4). The OR for sCD27 was changed from 0.94 (95% CI: 0.68–1.31) to 0.55 (95% CI: 0.32–0.96).

**TABLE 2:** The mean values (standard deviation) of circulating protein levels in cases and controls

Protein, unit	Case, mean (standard deviation)			Control, mean (standard deviation)		
	Single sample	First sample	Repeated sample	Single sample	First sample	Repeated sample
IL‐13, pg/ml	19.29 (22.98)	33.15 (33.09)	37.96 (59.83)	29.81 (31.55)	31.82 (37.08)	27.76 (23.54)
MCP‐3, pg/ml	90.20 (65.47)	69.55 (51.08)	76.99 (107.98)	117.23 (89.95)	64.86 (47.93)	61.67 (32.34)
MIP‐1α, pg/ml	15.64 (10.46)	22.61 (79.40)	14.17 (21.31)	19.20 (11.77)	9.99 (5.38)	9.57 (5.59)
MIP‐1β, pg/ml	44.45 (20.75)	58.44 (94.29)	49.61 (41.22)	49.55 (29.18)	45.88 (32.51)	42.11 (23.46)
TGF‐α, pg/ml	5.68 (5.72)	6.21 (13.55)	9.27 (27.8)	9.10 (10.37)	5.17 (4.75)	5.14 (4.33)
VEGF, pg/ml	456.46 (320.54)	486.14 (485.10)	475.83 (539.19)	479.44 (317.36)	451.12 (478.39)	410.69 (309.39)
TNF‐α, pg/ml	15.96 (9.34)	14.73 (12.55)	14.21 (11.82)	19.17 (20.83)	13.26 (6.63)	13.08 (8.64)
FGF2, pg/ml	296.67 (119.89)	280.67 (159.1)	289.78 (253.29)	352.05 (209.9)	281.87 (267.36)	262.71 (190.21)
Fractalkine, pg/ml	427.64 (230.43)	350.28 (173.62)	370.87 (233.56)	491.98 (331.14)	343.03 (141.39)	334.63 (170.23)
IL‐10, pg/ml	26.01 (42.03)	14.34 (13.70)	15.51 (16.31)	29.73 (26.29)	14.93 (13.19)	15.52 (14.27)
sIL‐2Rα, pg/ml	386.21 (264.52)	451.37 (193.49)	494.19 (219.26)	386.77 (284.17)	420.08 (189.81)	464.84 (253.39)
sIL‐6R, pg/ml	14341.12 (7885.95)	16448.92 (4568.74)	17431.1 (7123.94)	14149.17 (7857.58)	15391.29 (5132.15)	16102.7 (5775.07)
sTNFR2, pg/ml	4803.48 (2237.9)	5438.13 (1590.76)	5830.95 (1682.5)	4734.47 (2225.93)	5113.15 (1399.89)	5516.35 (1846.77)
sVEGFR2, pg/ml	11597.09 (6609.52)	13445.82 (4883.64)	14460.24 (7966.89)	11604.29 (6417.34)	13507.76 (5176.34)	12961.18 (4318.31)
CXCL13, pg/ml	75.96 (63.59)	73.77 (38.35)	80.00 (61.15)	76.89 (49.54)	67.84 (25.44)	80.07 (83.92)
sTNFR1, pg/ml	4083.12 (1140.52)	3451.8 (942.81)	3856.27 (1064.98)	4215.92 (1352.89)	3383.54 (825.79)	3701.76 (856.71)
sCD23, pg/ml	4829.69 (4663.94)	2453.46 (1379.79)	2583.63 (1405.97)	5481.06 (6377.81)	2328.62 (1260.01)	2363.39 (1159.21)
sCD27, U/ml	45.14 (79.86)	18.40 (7.66)	22.29 (11.39)	37.41 (54.53)	19.71 (9.68)	21.04 (11.41)
sCD30, ng/ml	2.63 (1.32)	2.97 (1.39)	4.06 (8.05)	2.75 (1.33)	3.00 (1.61)	3.64 (6.29)

Abbreviations: IL‐13, interleukin 13; MCP‐3, monocyte chemoattractant protein 3; MIP‐1α, macrophage inflammatory protein‐1 alpha; MIP‐1β, macrophage inflammatory protein‐1 beta; TGF‐α, transforming growth factor alpha; VEGF, vascular endothelial growth factor; TNF‐α, tumour necrosis factor alpha; FGF2, fibroblast growth factor 2; IL‐10, interleukin 10, sIL‐2Rα, soluble interleukin 2 receptor alpha; sIL‐6R, soluble interleukin 6 receptor; sTNFR2, soluble tumour necrosis factor receptor 2; sVEGFR2, soluble vascular endothelial growth factor receptor 2; CXCL13, chemokine C‐X‐C motif ligand 13; sTNFR1, soluble tumour necrosis factor receptor 1; sCD23, soluble CD23; sCD27, soluble CD27; sCD30, soluble CD30.

**TABLE 3:** Association between pre‐diagnostic levels of proteins and risk of glioma

Protein	Crude model			Adjusted model^a^		
	OR	95% CI	*p*‐value	OR	95% CI	*p*‐value
IL‐13	0.86	(0.72, 1.02)	0.088	0.86	(0.72, 1.02)	0.086
MCP‐3	0.90	(0.68, 1.20)	0.482	0.90	(0.68, 1.20)	0.483
MIP‐1α	0.90	(0.70, 1.16)	0.419	0.90	(0.70, 1.16)	0.414
MIP‐1β	1.08	(0.84, 1.39)	0.553	1.08	(0.84, 1.39)	0.555
TGF‐α	0.92	(0.77, 1.11)	0.396	0.92	(0.76, 1.12)	0.406
VEGF	0.89	(0.69, 1.15)	0.376	0.89	(0.69, 1.15)	0.376
TNF‐α	1.01	(0.75, 1.37)	0.950	1.01	(0.74, 1.37)	0.951
FGF2	0.98	(0.73, 1.31)	0.877	0.98	(0.73, 1.31)	0.877
Fractalkine	0.93	(0.69, 1.24)	0.620	0.93	(0.69, 1.25)	0.622
IL‐10	0.97	(0.84, 1.12)	0.689	0.97	(0.84, 1.12)	0.688
sIL‐2Rα	1.47	(1.01, 2.14)	0.045	1.48	(1.01, 2.16)	0.044
sIL‐6R	1.89	(1.14, 3.16)	0.014	1.90	(1.14, 3.17)	0.014
sTNFR2	1.71	(1.01, 2.90)	0.047	1.72	(1.01, 2.93)	0.045
sVEGFR2	1.28	(0.72, 2.25)	0.401	2.44^b^	(1.29, 4.61)	0.006
CXCL13	1.09	(0.74, 1.62)	0.663	1.09	(0.74, 1.62)	0.662
sTNFR1	1.21	(0.61, 2.42)	0.589	1.24	(0.59, 2.60)	0.576
sCD23	1.18	(0.75, 1.85)	0.473	1.18	(0.75, 1.85)	0.472
sCD27	0.94	(0.68, 1.30)	0.719	0.94	(0.68, 1.31)	0.729
sCD30	1.12	(0.76, 1.65)	0.560	1.12	(0.76, 1.65)	0.560

Abbreviations: IL‐13, interleukin 13; MCP‐3, monocyte chemoattractant protein 3; MIP‐1α, macrophage inflammatory protein‐1 alpha; MIP‐1β, macrophage inflammatory protein‐1 beta; TGF‐α, transforming growth factor alpha; VEGF, vascular endothelial growth factor; TNF‐α, tumour necrosis factor alpha; FGF2, fibroblast growth factor 2; IL‐10, interleukin 10; sIL‐2Rα, soluble interleukin 2 receptor alpha; sIL‐6R, soluble interleukin 6 receptor; sTNFR2, soluble tumour necrosis factor receptor 2; sVEGFR2, soluble vascular endothelial growth factor receptor 2; CXCL13, chemokine C‐X‐C motif ligand 13; sTNFR1, soluble tumour necrosis factor receptor 1; sCD23, soluble CD23; sCD27, soluble CD27; sCD30, soluble CD30; OR, odds ratio; CI, confidence interval

^a^ The models were adjusted for sample collection time defined by the years before the date of diagnosis amongst cases and corresponding matched time for controls.

^b^ The risk of glioma on sVEGFR2 was modified by blood sample collected time (*p* for interaction: 0.015). The odds ratio is 2.44×e*^0^* ^.^ *^11^* ^×time^.

## 4 DISCUSSION

The investigation of blood biomarkers in repeated pre‐diagnostic samples is a powerful tool for finding pathways of proteins that can explain the trajectories of disease development and for finding the potential aetiological risk factors. Taking advantage of the longitudinal design of the NSHDS, we found that the pre‐diagnostic plasma levels for sVEGFR2, sTNFR2, sIL‐2Rα and sIL‐6R were associated with glioma risk, a finding that suggests these proteins might play a role in glioma development.

### 4.1 sVEGFR2

Angiogenesis is an important feature in glioblastoma growth, especially in the early stage of tumour development. VEGF and VEGFR2 are important mediators of angiogenesis and highly expressed in GBM.^15^ VEGF and VEGFR2 seem to be important in the regulation of the innate immune system in the brain by microglia and macrophages.^16^ Several studies have targeted the ‘VEGF–VEGFR pathway for therapy, for example, with bevacizumab, which alleviates some symptoms although does not change the overall survival.^17^ Imaging studies have been able to differentially visualise VEGFR2 to enable targeted therapy.^18^ The serum levels of VEGF, sVEGFR1 and sVEGFR2 have also been used to monitor the treatment effect.^19^ In a nested case–control study, Schwartzbaum et al. found that elevated pre‐diagnostic levels of VEGF are associated with increased risk of glioma.^12^ However, we as well as Brenner et al. were unable to confirm this finding.^10^ Interestingly, we found that the sVEGFR2 was associated with glioma risk, but sVEGFR2 was not included in Schwartzbaum et al.’s or Brenner et al.’s study. In our study, the correlation between VEGF and sVEGFR2 was low (Figure 2). Genetic variants in VEGFR2 have been associated with survival in GBM patients, but this association could not be confirmed in independent data sets.^20^ In addition, we did not find any genetic variants for glioma risk in VEGFR2 in comprehensive genome‐wide association analyses.^1^, ^20^ Our findings suggest that the sVEGFR2 might be involved in the development of glioma and reflect the differences in aetiological mechanisms between the ageing cases and the controls or the presence of a preclinical tumour, but future studies are needed to confirm the findings and elucidate the biological mechanisms.

### 4.2 sTNFR2

Tumour necrosis factor (TNF‐α) mediates immune and inflammatory responses by binding to TNF receptor 1 (TNFR1) and 2 (TNFR2). Compared with TNFR1, which is ubiquitously expressed, TNFR2 expression is restricted to immune cells and some other cell types, such as endothelial cells, cardiomyocytes and glial cells.^21^ TNF–TNFR2 interaction generally leads to immune suppression, and TNFR2 has been found to be highly expressed in regulatory T cells (Tregs) in human peripheral blood.^22^ In addition, elevated serum level of sTNFR2 is associated with various cancers.^23^, ^24^, ^25^ In a case–control study, serum concentrations of sTNFR2 were higher in recurrent GBM than in controls but were not significantly different from newly diagnosed GBM. In contrast, lower levels of sTNFR1 were observed in patients with newly diagnosed or recurrent GBM than in controls.^25^ In the current study, pre‐diagnostic plasma levels of sTNFR2 were significantly associated with increased risk of glioma, but sTNFR1 was not. These findings were robust when we restricted to the GBM subgroup in the sensitivity analysis. The levels of sTNFR1 were increased significantly over time both in cases and controls (Table S3); however, there was no difference between cases and controls. These changes in sTNFR1 might reflect changes in proteins due to ageing or other factors (e.g. the storage process) rather than the glioma risk.^26^

### 4.3 sIL‐2Rα

The association between pre‐diagnostic levels of plasma sIL‐2Rα and glioma risk is plausible considering the biological evidence in the literature. IL2‐Rα (also called CD25) plays a critical role in the development and maintenance of Tregs.^27^ The accumulation of CD4^+^CD25^+^Foxp3^+^ Tregs is one of the hallmark features of GBM, and Tregs have been the predominant targets for immunotherapy in glioma models and patients.^28^, ^29^ The increased concentrations of sIL‐2Rα in the blood are likely to be the result of activation of normal peripheral mononuclear cells in response to the neoplasm's growth or of activated lymphoid cells infiltrating neoplastic tissues.^30^ Elevated serum levels of sIL‐2Rα have been observed in patients with diverse diseases (e.g. autoimmune, inflammatory and neoplastic diseases)^30^ and are related to glioma recurrence.^31^ However, a previous study did not find a significant association between sIL‐2Rα and the glioma risk in pre‐diagnostic serum sample.^12^ We found a weaker and not significant association between sIL‐2Rα and GBM; however, the similar patterns of sIL‐2Rα in GBMs suggest the difference might be due to our small sample size.

### 4.4 sIL‐6R

Interleukin 6 (IL‐6) regulates diverse physiological functions that support gliomagenesis including cell invasion and migration by activating the JAK/STAT pathway.^32^ The complex of IL‐6 and sIL‐6R can bind to glycoprotein 130 on cells and it can drive the trans‐signalling process, which leads to the development of pro‐inflammatory responses.^32^ Interestingly, high expression levels of IL‐6 and IL‐6R have been associated with aggressive subtypes of glioma (i.e. mesenchymal subtype and IDH wild‐type glioma).^33^ Serum/plasma levels of sIL‐6R have been found to be related to several diseases but not to glioma, and the biological significance of sIL‐6R on the disease progression remains unclear.^34^ However, the results of this study show that increased plasma levels of sIL‐6R are associated with increased risk of glioma and the levels of sIL‐6R were constantly higher in cases than in controls, suggesting an aetiological role of sIL‐6R in glioma risk, especially for GBM.

### 4.5 sCD27

sCD27, a 32 kDa protein, is released after lymphocyte activation by splicing from membrane‐bound CD27, which is a glycosylated transmembrane protein of the TNF receptor family.^35^ Bound to its ligand CD70, CD27–CD70 interactions play an important role in enhancing T‐cell proliferation and differentiation and therefore is a potential target in cancer immunotherapy.^36^ Varlilumab, an CD27 agonist, is now being administered in ongoing clinical trials for several cancers, including gliomas.^36^ Like Schwartzbuam et al., ^12^ we found no significant association between pre‐diagnostic levels of sCD27 and glioma. Interestingly, we did find lower level of sCD27 in the pre‐diagnostic samples of GBMs, which might reflect the heterogeneity of gliomas. Similar results were found when the sCD27 was analysed by Luminex bead‐based multiplex assays (LXSAHM kit, R&D Systems, USA). The ORs changed from 0.66 (95% CI = 0.37–1.19) to 0.33 (95% CI = 0.17–0.67) for gliomas and GBM, respectively. However, these findings may be somewhat limited by the small sample sizes in the sensitivity analysis.

### 4.6 Comparison with other studies

The association between pre‐diagnostic protein levels and glioma has been investigated in two other large prospective cohort studies.^10^, ^11^, ^12^ In line with our findings, previous cohort studies found no association between glioma risk and levels of IL‐13, TNF‐α, CXCL13, MIP‐1α, MIP‐1β, fractalkine, FGF2, TGF‐α, sCD23, sCD27 and sCD30.^10^, ^11^, ^12^ Neither of these studies included sVEGFR2 and sTNFR2 in their panel of investigated markers. Schwartzbaum et al. did not find a significant association between sIL‐2Rα or sIL‐6R and glioma. The four proteins discussed above were not investigated in Brenner et al.^10^ In general, differences between the panels of investigated markers, study design and statistical and laboratory methods make it difficult to compare our results with Schwartzbaum et al.’s and Brenner et al.’s results. As in our study, Brenner et al. analysed protein levels in multiple samples taken at different times from the same individual. However, in their large cohort of military personnel, the large majority of identified cases were low‐grade glioma diagnosed in males younger than 40 years old. However, in our study, where the first blood sample was collected at a median age of 50 years, the median age of diagnosis was 62 years and the majority of cases were diagnosed with a high‐grade glioma. Our study's age at diagnosis and blood sampling were more similar to Schwartzbaum et al.’s study, which includes population‐based samples from the Janus cohort in Norway, although the Norwegian study is limited to a single sample from each subject.

One of the potential limitations of our study is the limited number of cases, which is often a problem when studying glioma as obtaining sufficient numbers of pre‐diagnostic samples is difficult due to the rarity of glioma. Furthermore, we assumed a linear relationship between proteins and sample collection times when investigating protein trajectories. The true relationship might be more complicated. Although it is interesting to investigate how the cytokine and their soluble receptors act together to affect glioma risk, the multivariable models were not performed due to the small sample sizes. We did not find a strong correlation between cytokines and their receptors. As we performed several comparisons, this could have introduced the probability of chance findings. It should be noted that our findings are not statistically significant after considering the multiple testing correction. However, evidence from the literature supports plausible roles of these four proteins in gliomagenesis. In addition, the cytokines interact with each other and with specific receptors so the concentrations of an individual cytokine or receptor might not be biologically meaningful.^12^ Further studies will be required to validate our findings.

In conclusion, we selected 19 proteins that have been significantly linked to haematological cancers and in experimental studies associated with glioma growth. Most cytokines were not significantly associated with glioma. However, we found that four proteins––sVEGFR2, sTNFR2, sIL‐2Rα and sIL‐6R––might play important roles in the development of glioma. Future studies should explore the genetic and tumour differences in patients with pre‐diagnostic high levels of these cytokines, especially their underlying functionality.

## ETHICS STATEMENT

The study was conducted according to the guidelines of the Declaration of Helsinki and approved by the Regional Ethical Review Board at Umeå University, Umeå Sweden (Ethical approval number 2017‐295‐31M and 2018‐87‐32M). Informed consent was obtained from all subjects involved in the study.

## CONFLICT OF INTEREST

The authors declare no conflict of interest.

## AUTHORS CONTRIBUTIONS

Conceptualisation, B.M., F.S. and W.Y‐Y.W.; methodology, W.Y‐Y.W., A.M.D. and C.W.; software, W.Y‐Y.W. and C.W.; formal analysis, W.Y‐Y.W. and A.M.D.; resources, B.M. and F.S.; data curation, F.S. and W.Y‐Y.W.; writing—original draft preparation, W.Y‐Y.W. and A.M.D.; writing—review and editing, all authors; visualisation, W.Y‐Y.W., A.M.D. and C.W.; supervision, B.M.; project administration, B.M.; funding acquisition, B.M. All authors have read and agreed to the published version of the manuscript.

## Supporting information

Supplementary Materials

## Data Availability

The data presented in this study are available upon request from the corresponding author.

## References

[1] Melin BS , Barnholtz‐Sloan JS , Wrensch MR , et al. Genome‐wide association study of glioma subtypes identifies specific differences in genetic susceptibility to glioblastoma and non‐glioblastoma tumors. Nat Genet. 2017;49:789‐794. 28346443 10.1038/ng.3823 PMC5558246

[2] Molinaro AM , Taylor JW , Wiencke JK , Wrensch MR . Genetic and molecular epidemiology of adult diffuse glioma. Nat Rev Neurol. 2019;15:405‐417. 31227792 10.1038/s41582-019-0220-2 PMC7286557

[3] Amirian ES , Zhou R , Wrensch MR , et al. Approaching a scientific consensus on the association between allergies and glioma risk: a report from the glioma international case‐control study. Cancer Epidemiol Biomarkers Prev. 2016;25:282‐290. 26908595 10.1158/1055-9965.EPI-15-0847 PMC4874516

[4] Galvao RP , Zong H . Inflammation and gliomagenesis: bi‐directional communication at early and late stages of tumor progression. Curr Pathobiol Rep. 2013;1:19‐28. 23538742 10.1007/s40139-012-0006-3 PMC3607461

[5] Iwami K , Natsume A , Wakabayashi T . Cytokine networks in glioma. Neurosurg Rev. 2011;34:253‐263; discussion 63‐4. 21656131 10.1007/s10143-011-0320-y

[6] Gieryng A , Pszczolkowska D , Walentynowicz KA , Rajan WD , Kaminska B . Immune microenvironment of gliomas. Lab Invest. 2017;97:498‐518. 28287634 10.1038/labinvest.2017.19

[7] Vajdic CM , Falster MO , de Sanjose S , et al. Atopic disease and risk of non‐Hodgkin lymphoma: an InterLymph pooled analysis. Cancer Res. 2009;69:6482‐6489. 19654312 10.1158/0008-5472.CAN-08-4372 PMC2758272

[8] Spath F , Wibom C , Krop EJ , et al. Biomarker dynamics in B‐cell lymphoma: a longitudinal prospective study of plasma samples up to 25 years before diagnosis. Cancer Res. 2017;77:1408‐1415. 28108506 10.1158/0008-5472.CAN-16-2345

[9] Purdue MP , Lan Q , Langseth H , Grimsrud TK , Hildesheim A , Rothman N . Prediagnostic serum sCD27 and sCD30 in serial samples and risks of non‐Hodgkin lymphoma subtypes. Int J Cancer. 2020;146:3312‐3319. 31523805 10.1002/ijc.32684 PMC10123845

[10] Brenner AV , Inskip PD , Rusiecki J , Rabkin CS , Engels J , Pfeiffer RM . Serially measured pre‐diagnostic levels of serum cytokines and risk of brain cancer in active component military personnel. Br J Cancer. 2018;119:893‐900. 30297770 10.1038/s41416-018-0272-x PMC6189110

[11] Schwartzbaum J , Seweryn M , Holloman C , et al. Association between prediagnostic allergy‐related serum cytokines and glioma. PLoS One. 2015;10:e0137503. 26352148 10.1371/journal.pone.0137503 PMC4564184

[12] Schwartzbaum J , Wang M , Root E , et al. A nested case‐control study of 277 prediagnostic serum cytokines and glioma. PLoS One. 2017;12:e0178705. 28594935 10.1371/journal.pone.0178705 PMC5464586

[13] Therneau T . A package for survival analysis in R. R package version 3.2‐7, 2020.

[14] Bates D , Mächler M , Bolker B , Walker S . Fitting linear mixed‐effects models using lme4. J Stat Softw. 2015;67:48.

[15] Loureiro LVM , Neder L , Callegaro‐Filho D , de Oliveira KL , Stavale JN , Malheiros SMF . The immunohistochemical landscape of the VEGF family and its receptors in glioblastomas. Surg Exp Pathol. 2020;3:9.

[16] Turkowski K , Brandenburg S , Mueller A , et al. VEGF as a modulator of the innate immune response in glioblastoma. Glia. 2018;66:161‐174. 28948650 10.1002/glia.23234

[17] Ameratunga M , Pavlakis N , Wheeler H , Grant R , Simes J , Khasraw M . Anti‐angiogenic therapy for high‐grade glioma. Cochrane Database Syst Rev. 2018;11:CD008218. 30480778 10.1002/14651858.CD008218.pub4 PMC6516839

[18] Mitran B , Guler R , Roche FP , et al. Radionuclide imaging of VEGFR2 in glioma vasculature using biparatopic affibody conjugate: proof‐of‐principle in a murine model. Theranostics. 2018;8:4462‐4476. 30214632 10.7150/thno.24395 PMC6134937

[19] Batchelor TT , Gerstner ER , Emblem KE , et al. Improved tumor oxygenation and survival in glioblastoma patients who show increased blood perfusion after cediranib and chemoradiation. Proc Natl Acad Sci U S A. 2013;110:19059‐19064. 24190997 10.1073/pnas.1318022110 PMC3839699

[20] Sjostrom S , Wibom C , Andersson U , et al. Genetic variations in VEGF and VEGFR2 and glioblastoma outcome. J Neurooncol. 2011;104:523‐527. 21191630 10.1007/s11060-010-0504-2 PMC3161189

[21] Medler J , Wajant H . Tumor necrosis factor receptor‐2 (TNFR2): an overview of an emerging drug target. Expert Opin Ther Targets. 2019;23:295‐307. 30856027 10.1080/14728222.2019.1586886

[22] Chen X , Subleski JJ , Hamano R , Howard OM , Wiltrout RH , Oppenheim JJ . Co‐expression of TNFR2 and CD25 identifies more of the functional CD4+FOXP3+ regulatory T cells in human peripheral blood. Eur J Immunol. 2010;40:1099‐1106. 20127680 10.1002/eji.200940022 PMC3096013

[23] Dossus L , Becker S , Rinaldi S , et al. Tumor necrosis factor (TNF)‐alpha, soluble TNF receptors and endometrial cancer risk: the EPIC study. Int J Cancer. 2011;129:2032‐2037. 21154749 10.1002/ijc.25840

[24] Mielczarek‐Palacz A , Kondera‐Anasz Z , Sikora J . Higher serum levels of tumour necrosis factor and its soluble receptors are associated with ovarian tumours. Arch Med Sci. 2012;8:848‐853. 23185194 10.5114/aoms.2012.31384 PMC3506230

[25] Ahluwalia MS , Bou‐Anak S , Burgett ME , et al. Correlation of higher levels of soluble TNF‐R1 with a shorter survival, independent of age, in recurrent glioblastoma. J Neurooncol. 2017;131:449‐458. 27858267 10.1007/s11060-016-2319-2 PMC5352462

[26] Hassan L , Medenwald D , Tiller D , et al. The association between change of soluble tumor necrosis factor receptor R1 (sTNF‐R1) measurements and cardiovascular and all‐cause mortality‐Results from the population‐based (Cardiovascular Disease, Living and Ageing in Halle) CARLA study 2002–2016. PLoS One. 2020;15:e0241213. 33104754 10.1371/journal.pone.0241213 PMC7588092

[27] Burchill MA , Yang J , Vang KB , Farrar MA . Interleukin‐2 receptor signaling in regulatory T cell development and homeostasis. Immunol Lett. 2007;114:1‐8. 17936914 10.1016/j.imlet.2007.08.005 PMC2094047

[28] Wainwright DA , Dey M , Chang A , Lesniak MS . Targeting tregs in malignant brain cancer: overcoming IDO. Front Immunol. 2013;4:116. 23720663 10.3389/fimmu.2013.00116 PMC3654236

[29] Ooi YC , Tran P , Ung N , et al. The role of regulatory T‐cells in glioma immunology. Clin Neurol Neurosurg. 2014;119:125‐132. 24582432 10.1016/j.clineuro.2013.12.004

[30] Bien E , Balcerska A . Serum soluble interleukin 2 receptor alpha in human cancer of adults and children: a review. Biomarkers. 2008;13:1‐26. 17906988 10.1080/13547500701674063

[31] Yoshida S , Morii K . Serum concentrations of soluble interleukin‐2 receptor in patients with malignant brain tumors. J Surg Oncol. 2000;75:131‐135. 11064393 10.1002/1096-9098(200010)75:2<131::aid-jso10>3.0.co;2-l

[32] West AJ , Tsui V , Stylli SS , et al. The role of interleukin‐6‐STAT3 signalling in glioblastoma. Oncol Lett. 2018;16:4095‐4104. 30250528 10.3892/ol.2018.9227 PMC6144698

[33] Jiang Y , Han S , Cheng W , Wang Z , Wu A . NFAT1‐regulated IL6 signalling contributes to aggressive phenotypes of glioma. Cell Commun Signal. 2017;15:54. 29258522 10.1186/s12964-017-0210-1 PMC5735798

[34] Jones SA , Horiuchi S , Topley N , Yamamoto N , Fuller GM . The soluble interleukin 6 receptor: mechanisms of production and implications in disease. FASEB J. 2001;15:43‐58. 11149892 10.1096/fj.99-1003rev

[35] van de Ven K , Borst J . Targeting the T‐cell co‐stimulatory CD27/CD70 pathway in cancer immunotherapy: rationale and potential. Immunotherapy. 2015;7:655‐667. 26098609 10.2217/imt.15.32

[36] Starzer AM , Berghoff AS . New emerging targets in cancer immunotherapy: CD27 (TNFRSF7). ESMO Open. 2020;4:e000629. 32152062 10.1136/esmoopen-2019-000629 PMC7082637

