# Supplementary material for: Pre‐diagnostic levels of sVEGFR2, sTNFR2, sIL‐2Rα and sIL‐6R are associated with glioma risk: A nested case–control study of repeated samples
Source: Cancer Med. 2022 Jan 14;11(4):1016–25. doi: 10.1002/cam4.4505 (PMC8855896; doi:10.1002/cam4.4505)
Supplement: Supplementary file 1 — Supplementary Materials [file CAM4-11-1016-s001.docx]

# Supplementary

**Table S1.** Percent above lower limit of detection (LLOD), and intra-assay coefficient of variation (CV) for measured proteins.

| **Protein** | **LLOD (%)** | **Intra-assay CV (%)** |
| --- | --- | --- |
| IL-13 | 93.92 | 21.49 |
| MCP-3 | 98.99 | 18.96 |
| MIP-1α | 97.72 | 20.08 |
| MIP-1β | 100.00 | 15.90 |
| TGF-α | 84.05 | 21.29 |
| VEGF | 98.99 | 19.79 |
| TNF-α | 100.00 | 13.50 |
| FGF-2 | 99.75 | 16.71 |
| Fractalkine | 100.00 | 18.04 |
| IL-10 | 95.19 | 17.24 |
| sIL-2Rα | 100.00 | 15.17 |
| sIL-6R | 100.00 | 8.31 |
| sTNFR2 | 100.00 | 10.93 |
| sVEGFR2 | 100.00 | 11.16 |
| CXCL13 | 99.75 | 12.86 |
| sTNFR1 | 99.75 | 9.50 |
| sCD23 | 100.00 | 12.63 |
| sCD27 | 99.24 | 24.01 |
| sCD30 ^a^ | 92.66 | - |

^a^ sCD30 was only measured once due to limited plasma volume.

**Table S2.** Body mass index (BMI) and fasting status of study subjects at time of blood sampling and thawing status of samples.

| **Characteristics** | **Cases, N** | **%** | **Controls, N** | **%** | ***p*-value ^a^** |
| --- | --- | --- | --- | --- | --- |
| BMI, median kg/m^2^ (± SD) |  |  |  |  |  |
| Single sample | 25.6 ± 3.7 |  | 26.0 ± 4.1 |  |  |
| First sample | 24.5 ± 3.2 |  | 25.1 ± 3.2 |  |  |
| Repeated sample | 25.4 ± 3.5 |  | 26.3 ± 3.4 |  |  |
| Smoking status, single sample |  |  |  |  |  |
| Non smoker | 27 | 39.7 | 19 | 27.9 |  |
| smoker | 10 | 14.7 | 13 | 19.1 |  |
| Former smoker | 19 | 27.9 | 24 | 35.3 |  |
| No information | 12 | 17.6 | 12 | 17.6 | 0.50 |
| Smoking status, first sample |  |  |  |  |  |
| Non smoker | 26 | 40.0 | 22 | 33.8 |  |
| smoker | 10 | 15.4 | 17 | 26.2 |  |
| Former smoker | 14 | 21.5 | 12 | 18.5 |  |
| No information | 15 | 23.1 | 14 | 21.5 | 0.51 |
| Smoking status, repeated sample |  |  |  |  |  |
| Non smoker | 20 | 30.8 | 14 | 21.5 |  |
| smoker | 7 | 10.8 | 6 | 9.2 |  |
| Former smoker | 4 | 6.2 | 11 | 16.9 |  |
| No information | 34 | 52.3 | 34 | 52.3 | 0.22 |
| Fasting status, single sample^b^ |  |  |  |  |  |
| 0-4 hours | 9 | 13.2 | 11 | 16.4 |  |
| 4-8 hours | 13 | 19.1 | 12 | 17.9 |  |
| >8 hours | 46 | 67.6 | 44 | 65.7 | 0.87 |
| Fasting status, first sample |  |  |  |  |  |
| 0-4 hours | 13 | 20.0 | 14 | 21.5 |  |
| 4-8 hours | 13 | 20.0 | 14 | 21.5 |  |
| >8 hours | 39 | 60.0 | 37 | 56.9 | 0.94 |
| Fasting status, repeated sample |  |  |  |  |  |
| 0-4 hours | 33 | 50.8 | 34 | 52.3 |  |
| 4-8 hours | 1 | 1.5 | 0 | 0.0 |  |
| >8 hours | 31 | 47.7 | 31 | 47.7 | 1.00 |
| Thawing status, single sample |  |  |  |  |  |
| No | 66 | 97.1 | 66 | 97.1 |  |
| Once | 2 | 2.9 | 2 | 2.9 | 1.00 |
| Thawing status, first sample |  |  |  |  |  |
| No | 60 | 92.3 | 61 | 93.8 |  |
| Once | 5 | 7.7 | 4 | 6.2 | 1.00 |
| Thawing status, repeated sample |  |  |  |  |  |
| No | 65 | 100.0 | 65 | 100.0 |  |
| Once | 0 | 0.0 | 0 | 0.0 | NA |

Abbreviation: BMI, body mass index; SD, standard deviation.

^a^ *P*-values were calculated using the chi-squared test or Fisher’s exact test (when the expected number in a single cell was < 5)

^b^ Information on fasting status at time of blood sampling was missing for one control individual.

**Table S3.** Association between disease status and protein levels over time based on the linear-mixed models in all samples and GBM subset.

| **Protein** | **Estimated parameter** | **All samples** | | | **GBM subset** | | |
| --- | --- | --- | --- | --- | --- | --- | --- |
|  |  | **Regression coefficient** | ***p*-value** | ***p*-value for interaction** | **Regression coefficient** | ***p*-value** | ***p*-value for interaction** |
| sVEGFR2 | Disease status | 0.099 | **0.0309** |  | 0.1003 | 0.075 |  |
|  | Time effect in controls | -0.0023 | 0.5081 |  | -0.0003 | 0.950 |  |
|  | Time effect in cases | **0.0082** | **0.0193** | **0.016** | **0.0082** | **0.043** | 0.096 |
| sIL-2Rα | Disease status | 0.0981 | 0.1432 |  | 0.0702 | 0.411 |  |
|  | Time effect in controls | 0.0081 | 0.1116 |  | 0.0101 | 0.101 |  |
|  | Time effect in cases | **0.0135** | **0.0077** | 0.396 | 0.0119 | 0.052 | 0.816 |
| sTNFR2 | Disease status | 0.0685 | 0.1586 |  | 0.0725 | 0.221 |  |
|  | Time effect in controls | 0.0067 | 0.0704 |  | **0.0082** | **0.044** |  |
|  | Time effect in cases | **0.0103** | **0.0052** | 0.44 | **0.0111** | **0.006** | 0.579 |
| sIL-6R | Disease status | 0.0405 | 0.4384 |  | 0.0740 | 0.283 |  |
|  | Time effect in controls | **0.0093** | **0.0178** |  | 0.0071 | 0.118 |  |
|  | Time effect in cases | 0.0075 | 0.0561 | 0.706 | 0.0071 | 0.113 | 0.995 |
| sTNFR1 | Disease status | 0.0115 | 0.7539 |  | -0.0065 | 0.891 |  |
|  | Time effect in controls | **0.0096** | **0.0001** |  | **0.0106** | **0.0004** |  |
|  | Time effect in cases | **0.0108** | **0.00002** | 0.728 | **0.0115** | **0.0001** | 0.824 |
| sCD27 | Disease status | 0.0384 | 0.6167 |  | -0.0871 | 0.338 |  |
|  | Time effect in controls | **0.0098** | **0.0357** |  | 0.0102 | 0.050 |  |
|  | Time effect in cases | **0.0169** | **0.0003** | 0.244 | **0.0140** | **0.007** | 0.577 |
| IL-13 | Disease status | -0.2804 | 0.0906 |  | -0.1590 | 0.419 |  |
|  | Time effect in controls | 0.0153 | 0.1584 |  | 0.0200 | 0.097 |  |
|  | Time effect in cases | 0.0051 | 0.6485 | 0.495 | 0.0139 | 0.263 | 0.714 |
| MCP-3 | Disease status | -0.0623 | 0.5166 |  | -0.0217 | 0.859 |  |
|  | Time effect in controls | 0.0011 | 0.8699 |  | 0.0076 | 0.323 |  |
|  | Time effect in cases | 0.0017 | 0.7917 | 0.942 | 0.0129 | 0.087 | 0.608 |
| MIP-1α | Disease status | -0.1284 | 0.3184 |  | -0.1147 | 0.469 |  |
|  | Time effect in controls | -0.0007 | 0.9391 |  | 0.0038 | 0.706 |  |
|  | Time effect in cases | -0.0038 | 0.6605 | 0.788 | 0.0016 | 0.870 | 0.875 |
| MIP-1β | Disease status | 0.0318 | 0.7738 |  | 0.0982 | 0.526 |  |
|  | Time effect in controls | -0.0053 | 0.4986 |  | -0.0047 | 0.648 |  |
|  | Time effect in cases | -0.0035 | 0.6544 | 0.864 | 0.0040 | 0.701 | 0.536 |
| TGF-α | Disease status | -0.0695 | 0.6963 |  | -0.1017 | 0.660 |  |
|  | Time effect in controls | 0.0152 | 0.2023 |  | 0.0228 | 0.106 |  |
|  | Time effect in cases | 0.0211 | 0.0782 | 0.714 | 0.0267 | 0.059 | 0.839 |
| VEGF | Disease status | -0.1388 | 0.2114 |  | -0.1220 | 0.366 |  |
|  | Time effect in controls | 0.0015 | 0.834 |  | 0.0068 | 0.419 |  |
|  | Time effect in cases | -0.009 | 0.2155 | 0.297 | -0.0025 | 0.763 | 0.421 |
| TNF-α | Disease status | -0.0327 | 0.7154 |  | 0.0079 | 0.946 |  |
|  | Time effect in controls | -0.0014 | 0.8253 |  | 0.0025 | 0.743 |  |
|  | Time effect in cases | -0.0041 | 0.5221 | 0.75 | 0.0031 | 0.689 | 0.959 |
| FGF-2 | Disease status | -0.0598 | 0.5168 |  | 0.0092 | 0.938 |  |
|  | Time effect in controls | 0.0034 | 0.5922 |  | 0.0058 | 0.466 |  |
|  | Time effect in cases | -0.0012 | 0.8559 | 0.596 | 0.0052 | 0.510 | 0.955 |
| Fractalkine | Disease status | -0.0328 | 0.69 |  | 0.0280 | 0.784 |  |
|  | Time effect in controls | 0.0022 | 0.7186 |  | 0.0061 | 0.396 |  |
|  | Time effect in cases | 0.0019 | 0.7523 | 0.972 | 0.0086 | 0.229 | 0.791 |
| IL-10 | Disease status | -0.1113 | 0.5648 |  | -0.0209 | 0.930 |  |
|  | Time effect in controls | 0.0092 | 0.4999 |  | 0.0144 | 0.325 |  |
|  | Time effect in cases | 0.0079 | 0.5562 | 0.943 | 0.0225 | 0.122 | 0.687 |
| CXCL13 | Disease status | -0.0563 | 0.4422 |  | -0.0700 | 0.4531 |  |
|  | Time effect in controls | 0.0038 | 0.4134 |  | 0.0032 | 0.568498 |  |
|  | Time effect in cases | -0.0042 | 0.375 | 0.215 | -0.0044 | 0.426536 | 0.3209 |
| sCD23 | Disease status | 0.0228 | 0.7408 |  | -0.0657 | 0.461 |  |
|  | Time effect in controls | 0.0006 | 0.8828 |  | 0.0062 | 0.200 |  |
|  | Time effect in cases | 0.0018 | 0.6673 | 0.834 | 0.0011 | 0.815 | 0.436 |
| sCD30 | Disease status | 0.0287 | 0.6854 |  | 0.0745 | 0.464 |  |
|  | Time effect in controls | 0.0008 | 0.8774 |  | 0.0009 | 0.894 |  |
|  | Time effect in cases | 0.0036 | 0.503 | 0.69 | 0.0053 | 0.469 | 0.646 |

Regression coefficients with *p*-value<0.05 were highlighted in bold

**Table S4.** Association between pre-diagnostic levels of proteins and risk of glioblastoma

| **Protein** | **Crude model** | | | **Adjusted model ^a^** | | |
| --- | --- | --- | --- | --- | --- | --- |
|  | **OR** | **95% CI** | ***p*- value** | **OR** | **95% CI** | ***p*- value** |
| IL-13 | 0.91 | (0.72,1.15) | 0.424 | 0.91 | (0.72,1.15) | 0.414 |
| MCP-3 | 0.93 | (0.64,1.34) | 0.684 | 0.93 | (0.64,1.34) | 0.690 |
| MIP-1α | 0.87 | (0.65,1.17) | 0.345 | 0.87 | (0.65,1.16) | 0.340 |
| MIP-1β | 1.07 | (0.80,1.42) | 0.65 | 1.07 | (0.80,1.42) | 0.650 |
| TGF-α | 0.91 | (0.73,1.13) | 0.392 | 0.91 | (0.72,1.14) | 0.403 |
| VEGF | 0.91 | (0.66,1.26) | 0.562 | 0.91 | (0.66,1.26) | 0.563 |
| TNF-α | 1.05 | (0.72,1.52) | 0.815 | 1.05 | (0.72,1.52) | 0.814 |
| FGF-2 | 1.09 | (0.78,1.53) | 0.628 | 1.09 | (0.77,1.53) | 0.626 |
| Fractalkine | 1.05 | (0.73,1.51) | 0.788 | 1.05 | (0.73,1.52) | 0.780 |
| IL-10 | 0.99 | (0.82,1.19) | 0.912 | 0.99 | (0.82,1.19) | 0.907 |
| sIL-2Rα | 1.35 | (0.86,2.14) | 0.196 | 1.36 | (0.86,2.15) | 0.190 |
| sIL-6R | 2.30 | (1.22,4.34) | 0.010 | 2.30 | (1.22,4.36) | 0.010 |
| sTNFR2 | 2.04 | (1.02,4.06) | 0.044 | 2.08 | (1.03,4.18) | 0.040 |
| sVEGFR2 | 1.36 | (0.66,2.80) | 0.400 | 1.36 | (0.66,2.81) | 0.400 |
| CXCL13 | 1.02 | (0.62,1.69) | 0.929 | 1.02 | (0.62,1.69) | 0.927 |
| sTNFR1 | 0.84 | (0.36,1.98) | 0.689 | 0.84 | (0.33,2.12) | 0.709 |
| sCD23 | 0.93 | (0.55,1.55) | 0.774 | 0.93 | (0.55,1.56) | 0.781 |
| sCD27 | 0.56 | (0.33,0.96) | 0.036 | 0.55 | (0.32,0.96) | 0.037 |
| sCD30 | 1.24 | (0.76,2.03) | 0.396 | 1.24 | (0.76,2.03) | 0.380 |

^a^ The models were adjusted for sample collection time defined by the years before the date of diagnosis among cases and corresponding reference time for controls.
